# Reconstruction of metabolic pathways for the cattle genome

**DOI:** 10.1186/1752-0509-3-33

**Published:** 2009-03-12

**Authors:** Seongwon Seo, Harris A Lewin

**Affiliations:** 1 Institute for Genomic Biology, University of Illinois at Urbana-Champaign, Urbana, IL 61801, USA; 2 Department of Animal Sciences, University of Illinois at Urbana-Champaign, Urbana, IL 61801, USA

## Abstract

**Background:**

Metabolic reconstruction of microbial, plant and animal genomes is a necessary step toward understanding the evolutionary origins of metabolism and species-specific adaptive traits. The aims of this study were to reconstruct conserved metabolic pathways in the cattle genome and to identify metabolic pathways with missing genes and proteins. The MetaCyc database and PathwayTools software suite were chosen for this work because they are widely used and easy to implement.

**Results:**

An amalgamated cattle genome database was created using the NCBI and Ensembl cattle genome databases (based on build 3.1) as data sources. PathwayTools was used to create a cattle-specific pathway genome database, which was followed by comprehensive manual curation for the reconstruction of metabolic pathways. The curated database, CattleCyc 1.0, consists of 217 metabolic pathways. A total of 64 mammalian-specific metabolic pathways were modified from the reference pathways in MetaCyc, and two pathways previously identified but missing from MetaCyc were added. Comparative analysis of metabolic pathways revealed the absence of mammalian genes for 22 metabolic enzymes whose activity was reported in the literature. We also identified six human metabolic protein-coding genes for which the cattle ortholog is missing from the sequence assembly.

**Conclusion:**

CattleCyc is a powerful tool for understanding the biology of ruminants and other cetartiodactyl species. In addition, the approach used to develop CattleCyc provides a framework for the metabolic reconstruction of other newly sequenced mammalian genomes. It is clear that metabolic pathway analysis strongly reflects the quality of the underlying genome annotations. Thus, having well-annotated genomes from many mammalian species hosted in BioCyc will facilitate the comparative analysis of metabolic pathways among different species and a systems approach to comparative physiology.

## Background

Production of domesticated cattle (*Bos taurus *and *Bos indicus*) accounts for 7% of the total food consumption in the world [1] and contributes 17.0% of all farm cash receipts in the United States [2]. Thus, there has been a strong rationale for developing genomic resources that can be used to increase the rate of genetic improvement for milk and meat production, disease resistance, feed efficiency and reproductive performance. Understanding the biology of cattle, particularly the unique features of ruminant metabolism [3], is a prerequisite for the sustainability of the cattle industry. However, many gaps still exist in our understanding of ruminant metabolism and many other traits specific to cetartiodactyl mammals [4]. The recent sequencing of the cattle genome [5] provides the first opportunity to systematically link genetic and metabolic traits of cattle and other ruminants.

Genome-scale models are useful to analyze, interpret and predict the genotype-to-phenotype relationships in an organism [6]. Accordingly, there have been attempts to reconstruct genome-scale metabolic pathways for a variety of organisms, including bacteria [7], simple eukaryotes [8] and higher eukaryotes [9-11]. For example the Pathway Tools software package [12] has been used to generate organism-specific pathway genome databases (PGDBs) for bacteria [13], plants [14,15] and animals [9]. Using the PathoLogic algorithm [16], Pathway Tools computationally reconstructs organism-specific metabolic pathways and generates a new PGDB by matching the Enzyme Commission (EC) number and/or the name of the annotated gene product against enzymes in MetaCyc, a manually curated database containing over 900 pathways from more than 900 different organisms [17]. BioCyc  is a collection of more than 260 PGDBs generated using Pathway Tools followed by manual curation [18]. Among the mammals, PGDBs in BioCyc exist only for human and recently for mouse.

For the cattle reference genome assembly build 3.1, independent sets of gene models and annotations are available from the National Center for Biotechnology Information (NCBI) [19] and from Ensembl [20]. Both are dependent on sequence similarity of cattle proteins to homologs in other well-annotated organisms (e.g. human and mouse). Thus, there is now an opportunity to reconstruct bovine metabolism using these resources. For this, we developed an amalgamated cattle genome database from the NCBI and Ensembl gene models that incorporates all the available functional annotation information for cattle genes and proteins from other data sources.

Metabolic pathways were then identified using Pathway Tools and the reconstructed pathways of cattle were compared to those of other organisms. We also corrected and updated mammalian-specific metabolic pathways in MetaCyc, and identified enzymes not associated to genes.

## Results

### The amalgamated cattle genome annotation database

At the time of the present analysis, 28,732 and 25,132 genes in the cattle genome were predicted in the NCBI and Ensembl genome databases, respectively. For the two gene sets only 2,109 genes had exactly the same gene coordinates, and 6,479, 16,163, 7,026 and 1,360 genes had a common gene symbol, Entrez-Gene ID, gene product name, or EC number, respectively (Table 1).

**Table 1:** The number of consensus cattle gene pairs in the NCBI and Ensembl cattle genome databases

Type of match	Number of pairs^†^	Unique matches^‡^
Same gene coordinates	2,109	2,109
Gene symbol	6,479	5,187
Entrez gene ID	16,163	8,800
Gene product name	7,026	71
E.C. number	1,360	6
Manual*	N.A.	27
Total	33,137	16,200
Not matched	2,276	2,276

^†^The total number of matches using each criterion; one-to-one match was not assumed.

^‡^Number in each row is sequential for the match criteria given under "number of pairs", in addition to the matches for the criteria immediately above; only one-to-one matches were considered.

*Manually matched because the consensus genes were classified into different 'gene type' (e.g. protein coding, pseudogene, tRNA and miscellaneous RNA) in the NCBI and Ensembl gene models.

By sequential one-to-one matching, a total of 16,173 consensus gene models were identified. A total of 2,109 genes had exactly the same gene coordinates; the rest of the matching criteria sequentially identified 5,187 (gene symbol), 8,800 (Entrez-Gene ID), 71 (gene product name) and 6 (EC number) consensus gene pairs (Table 1).

When Entrez-Gene ID was used as the last matching criterion in the matching sequence, no difference in the total number of consensus genes was observed. Among the gene pairs that shared some portion of their gene coordinates and had the same "gene type" and coding strand, 2,276 were not considered as matches on the basis of the remaining matching criteria. During the manual curation of cattle PGDB, 27 gene pairs with overlapping coordinates that were classified as a different "gene type" in the NCBI and Ensembl databases were added back to cattle PGDG as consensus gene pairs. The amalgamated cattle genome database thus contains 16,200 (16,173 + 27) consensus cattle genes and has 12,287 and 8,932 genes contained exclusively in NCBI build 3.1 or Ensembl build 3.1, respectively (Table 2). In addition, 245 genes from NCBI genome scaffolds that were not incorporated into genome build 3.1 were included in the final build of the amalgamated cattle genome database.

**Table 2:** Distribution of genes in the amalgamated cattle genome database according to the original data sources

Source	Number of genes
Consensus build 3.1 (this study)	16,200
NCBI build 3.1 only	12,287
Ensembl build 3.1 only	8,932
NCBI other genome scaffolds*	245

Total	37,664

*Scaffolds not incorporated into NCBI genome build 3.1, including some scaffolds from the reference assembly build 2.1.

Amalgamated databases were also constructed for human, mouse and dog. The sequential matching process identified a total of 19,354, 20,118 and 14,147 genes in the NCBI and Ensembl databases for human, mouse and dog, respectively [see Additional file 1].

### Metabolic reconstruction of the cattle genome

**Figure 1:**
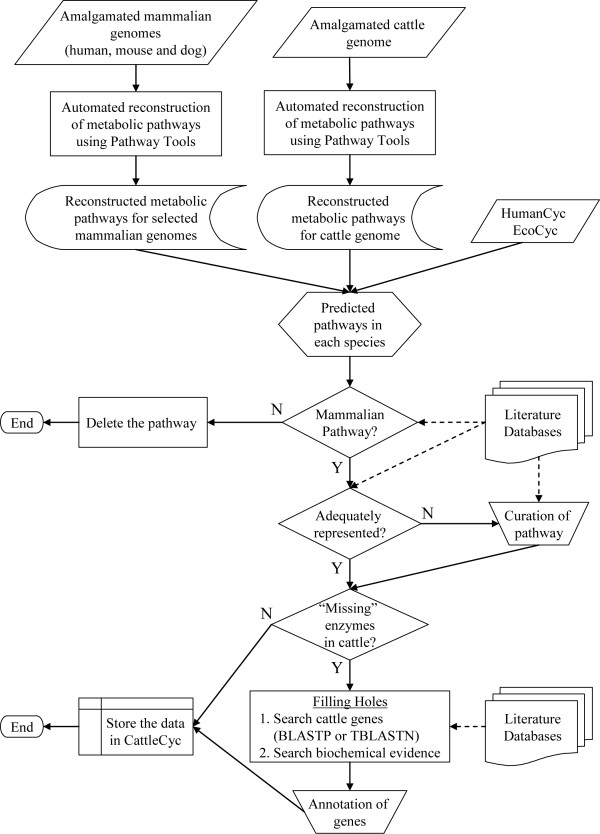
**The metabolism-centered approach used for the identification of metabolic pathways and "missing" enzymes**. Solid and dashed arrows represent data and information flows, respectively.

The general scheme of the metabolism-centered approach used for metabolic reconstruction of the cattle genome is shown in Figure 1. The initial automated construction of cattle PGDB using the PathoLogic algorithm recognized 1,008 and 164 enzymes (gene products) by EC number and gene product name matching, respectively. These were involved in 873 unique enzymatic reactions. The initial build of the cattle PGDB contained 243 metabolic pathways, 1,528 reactions, including 1,500 enzymatic, 25 spontaneous and 3 transport reactions, and 1,116 compounds (Table 3). An enzymatic reaction was defined as a chemical reaction that involves a single enzyme or an enzyme complex but does not mediate molecular transport. Because not all enzymatic reactions were incorporated into metabolic pathways, 1,059 out of 1,528 reactions and 473 out of 1,172 genes were present in the initial build of the cattle PGDG metabolic pathways. As shown in Table 3, 184 metabolic pathways contained one or more pathway holes, which are defined as reactions in which the organism-specific enzyme has not yet been identified. The total number of pathway holes was 593, or 56% of the total known reactions in pathways.

**Table 3:** Comparison of selected organism-specific pathway genome databases (PGDB)

Database statistics	Cattle		New PGDB without manual curation			BioCyc	
			
	Initial	Curated	Human	Mouse	Dog	E. coli	Human
Metabolic pathways	243	217	342	324	151	194	178
Enzymatic reactions	1,500	1,419	2,020	1,941	1,102	1,245	1,253
Enzymes	1,172	1,544	2,846	2,789	753	1,323	2,594
Compounds	1,116	1,021	1,390	1,325	794	1,202	975
							
Pathway holes (missing enzymes)							
number of pathway holes	593	113	587	536	509	35	246
pathway holes as a percentage of total reactions in pathways	56%	14%	45%	43%	67%	5%	36%
pathways with no holes	59	165	134	128	17	169	67
pathways with 1 hole	51	28	64	65	30	15	35
pathways with 2 holes	32	11	40	41	22	7	37
pathways with 3 holes	21	1	36	25	15	3	15
pathways with 4 holes	14	6	18	18	15	0	11
pathways with >5 holes	66	6	50	47	52	0	13
total pathways with holes	184	52	208	196	134	25	111

For comparison, the same approach used for the initial metabolic reconstruction of the cattle genome (Figure 1) was used for metabolic reconstruction of the human, mouse and dog genomes. The automated reconstructions identified 342, 324 and 151 metabolic pathways for human, mouse and dog genomes, respectively (Table 3). The larger number of predicted metabolic pathways in human and mouse compared to dog is mainly because the current annotation of the human and mouse genomes is more extensive than that of the dog genome. A relatively large percentage of reactions in pathways are in pathway holes; 45% in human and 43% in mouse. For dog, 67% of genes encoding enzymes in known pathways were not identified in the current annotation.

To improve metabolic reconstruction of the cattle and other mammalian genomes we manually reviewed 553 metabolic pathways present in HumanCyc, EcoCyc and also predicted in the automated reconstructions for human, mouse and dog. Out of the 243 automatically reconstructed cattle pathways, 79 pathways were deleted because previous biochemical evidence for these pathways existed only in microbes or plants. Fifty-one reference pathways from MetaCyc were modified manually in CattleCyc because they did not adequately represent mammalian metabolic pathways according to literature sources. After curation, these were added to the cattle PGDB.

**Figure 2:**
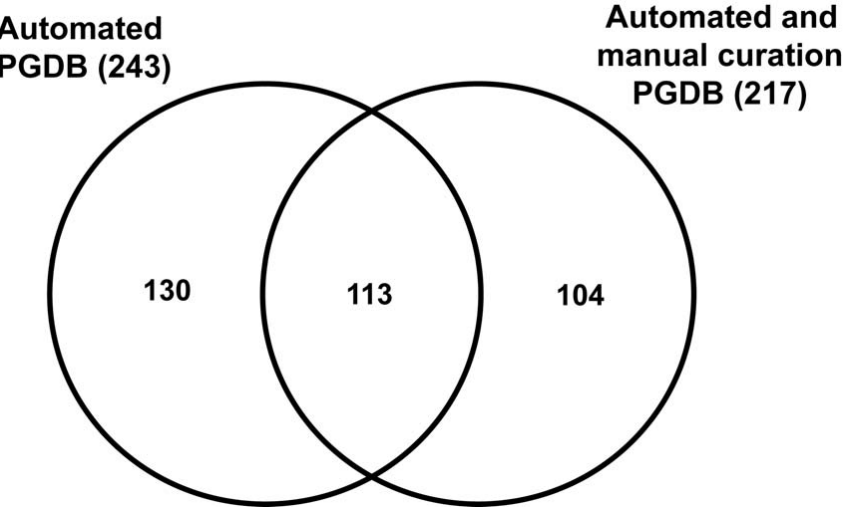
**Comparison of constructed metabolic pathways by automation with those after manual curation**. Numbers of metabolic pathways, shared by the cattle pathway genome database (PGDB) generated computationally and after manual curation. The numbers in parentheses are the total number of pathways in the corresponding cattle PGDB.

Additionally, 15 more mammalian metabolic pathways were created manually and 38 pathways from MetaCyc, which were not included in the initial reconstruction mainly due to incomplete annotation of the cattle genome, were also added manually. Consequently, the curated cattle PGDB contains 113 pathways from the automated reconstruction and 104 pathways that were manually added (Figure 2). A listing of the 66 new manually curated mammalian metabolic pathways created in CattleCyc is given [see Additional file 2].

The manually curated version of CattleCyc consists of 217 metabolic pathways that contain 736 genes involving 825 distinct enzymatic reactions. CattleCyc contains 1,544 enzymes in 1,277 known enzymatic reactions, 1,442 biochemical reactions including 1,419 enzymatic reactions, and 1,021 compounds (Table 3). At the time of writing the total number of genes having an annotated EC number in CattleCyc is 1,500, which is larger than 1,263 found in KEGG (Genome Database Release 07-07-26) and 1,346 in UniProt (Knowledgebase Release 12.0). A total of 113 pathway holes were present among 52 pathways in the manually curated version of the database. The total number of pathway holes as a percentage of total reactions in pathways is 14%, which is higher than EcoCyc (5%), but lower than the existing version of HumanCyc (36%).

Among 113 missing enzyme genes in the cattle metabolic pathways, the activities of six enzymes were reported in cattle (Table 4) [21-38]; 16 enzyme activities were reported in other mammals but not in cattle (Table 5) [39-67]. However, in both cases, corresponding mammalian genes have not been identified. Interestingly, no enzymatic activity for L-ascorbate peroxidase has been reported in any mammal, except for cattle. For six enzymes, the cattle orthologs of human genes *ECGF1*, *CERK*, *FAAH2*, *ALG12 *and *EARS2 *were not identified (Table 6). Neither a gene nor enzyme activity was identified for the other pathway holes; however, the pathways remain in the database because there is some evidence that they are present in mammals even though not all the reactions in the pathways have been validated.

**Table 4:** List of enzyme activities reported in cattle and other mammals that do not have a mammalian gene identified

Pathway	Enzyme name	EC number*	Mammalian species
Ascorbate glutathione cycle	L-ascorbate peroxidase^†^	1.11.1.11	cattle [21]
4-hydroxyproline degradation	4-hydroxy-2-oxoglutarate aldolase	4.1.3.16	cattle [27-30], rat [22]
Ascorbate biosynthesis	gluconolactonase	3.1.1.17	cattle [23,25], pig [35]
Ascorbate biosynthesis	uronolactonase	3.1.1.19	cattle [37], pig [37], rabbit [37], monkey [37], dog [37], guinea pig [37], rat [33,37]
γ-glutamyl cycle	γ-glutamyl cyclotransferase	2.3.2.4	cattle [26], human [38], rat [34], mouse [24], pig [36]
Selenocysteine biosynthesis	selenocysteine synthase	2.9.1.1	mouse [32], cattle [31]

*Enzyme Commission number

^†^Partial sequence of the protein is known (UniProt: Q7M3G0)

**Table 5:** List of metabolic enzyme activities without an identified mammalian gene

Pathway	Enzyme name	EC number*	Mammalian species
4-hydroxyproline degradation	4-oxoproline reductase	1.1.1.104	human [56], rabbit [56]
Ascorbate biosynthesis	glucuronolactone reductase	1.1.1.20	rat [48,54]
Ascorbate biosynthesis	1,4-lactonase	3.1.1.25	human [45,94], rat [45,94]
β-alanine biosynthesis	3-hydroxypropionate dehydrogenase	1.1.1.59	pig [43], chicken [43]
β-alanine biosynthesis	β-alanine-pyruvate transaminase	2.6.1.18	rat [49]
Cysteine degradataion	hypotaurine dehydrogenase	1.8.1.3	rat [57]
Degradation of purine deoxyribonucleosides	phosphopentomutase	5.4.2.7	human [58], rabbit [58], rat [40,58]
Glutathione detoxification	cycteine-S-conjugate N-acetyltransferase	2.3.1.80	human [47], rat [44,47], pig [39,51]
Histidine degradation	histidine aminotransferase	2.6.1.38	mouse [41]
Lysine degradation	pyrroline-2-carboxylate reductase	1.5.1.1	mouse [46], dog [46], rat [52]
Lysine degradation	L-lysine oxidase	1.4.3.14	mouse [53]
Purine degradation	guanosine deaminase	3.5.4.15	human [50], Rat [42]
Pyridine nucleotide cycling	nicotinamidase	3.5.1.19	mouse [59], rat [55], rabbit [55,60,61]
Tryptophan degradation	aminomuconate-semialdehyde dehydrogenase	1.2.1.32	cat [64]
UDP-N-acetylgalactosamine biosynthesis	UDP-N-acetylglucosamine 4-epimerase	5.1.3.7	human [62,67], rat [63], pig [66]
Valine degradation	pyruvate decarboxylase	4.1.1.1	human [65]

*Enzyme Commission number

**Table 6:** List of human genes with no cattle ortholog identified

Gene symbol	Gene Name	Refseq protein	EC number*	Entrez-Gene ID		
				
				Human	Mouse	Dog
*ECGF1*	endothelial cell growth factor 1 (platelet-derived)	NP_001944	2.4.2.4	1890	72962	none
*CERK*	ceramide kinase	NP_073603; NP_872602	2.7.1.138	64781	223753	474464
*FAAH2*	fatty acid amide hydrolase 2	NP_777572	3.5.1.4	158584	none	none
*ALG12*	asparagine-linked glycosylation 12 homolog	NP_077010	2.4.1.130	79087	223774	481196
*EARS2*	glutamyl-tRNA synthetase 2, mitochondrial (putative)	NP_001077083	6.1.1.17	124454	67417	479807

*Enzyme Commission number

**Figure 3:**
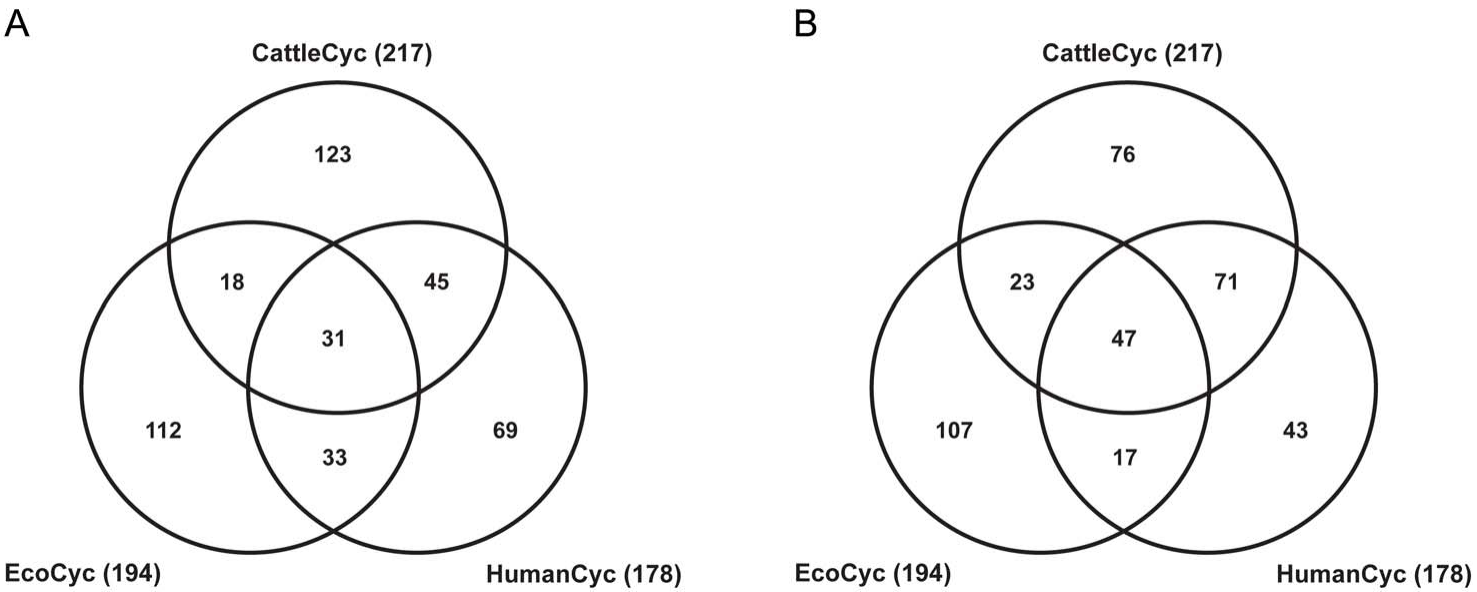
**Comparison of constructed 'Cyc' metabolic pathways among cattle, human and *E. coli***. Numbers of metabolic pathways, shared by CattleCyc 1.0, HumanCyc 11.0 and EcoCyc 11.0. The numbers in parentheses are the total number of pathways in the corresponding databases. (A) Consensus pathways at the enzyme level (all enzymes in the pathway have the same EC numbers). (B) Consensus pathways at the functional level (pathways that share biological function even though individual enzymes may differ and alternative reactions may exist).

The pathways contained in CattleCyc were compared with those in EcoCyc [13] and HumanCyc [9] (Figure 3). The consensus pathways among these databases were identified at both the enzyme (enzymes with the same EC numbers) and functional levels (a pathway that has the same biological function but individual enzymes may vary and alternative reactions may exist). Among the metabolic pathways contained in CattleCyc, EcoCyc and HumanCyc (Table 3), 31 and 47 pathways are shared at the enzyme and functional levels, respectively. There was one cattle-specific pathway identified (ascorbate biosynthesis), and a relatively small fraction of pathways were common between CattleCyc and HumanCyc (Figure 3). The limited degree of pathway sharing between the cattle and human databases is mainly because, despite intensive manual curation of HumanCyc [9], many pathways were deleted or manually revised in CattleCyc [see Additional file 3]. Comparative analysis of metabolic pathways in CattleCyc and EcoCyc indicates that enzymes involved in some pathways are highly conserved, including tRNA charging, nucleotide sugar biosynthesis, pyrimidine ribonucleotide biosynthesis, fatty acid β-oxidation and biosynthesis, glycogen degradation, coenzyme A biosynthesis, folate polyglutamylation, non-oxidative pentose phosphate pathway, and pyridoxal 5'-phosphate salvage pathway [see Additional file 4]. These pathways all involve more than five enzymatic reactions.

## Discussion

### The amalgamated cattle genome annotation database

There are collaborative efforts to identify a core set of protein coding regions that are consistently annotated in human and mouse [68]. Likewise, this is a goal of the Bovine Genome Sequencing Consortium [5]. Herein, we have attempted to resolve annotation discrepancies between NCBI and Ensembl for the cattle genome. In order to obtain a non-redundant gene set, HumanCyc [9] used Ensembl Build 31 as the main data source for annotation and merged Ensembl and Entrez genes if Ensembl included a cross reference to the Entrez-Gene ID. This approach, however, had a systematic problem when applied to the cattle genome. A total of 20,480 cattle Entrez-Gene ID were cross-referenced to 16,921 cattle genes in Ensembl. Out of these, 14,733 Ensembl genes had only one Entrez-Gene ID, whereas 1,649 and 539 genes contained 2 and >3 Entrez-Gene IDs, respectively. When each NCBI gene was paired with a corresponding Ensembl gene that had the same Entrez-Gene ID, a total of 20,443 gene pairs were obtained. Among those gene pairs, "gene type" and "coding strand" were not matched between NCBI and Ensembl for 1,245 and 1,693 cattle gene pairs, respectively. Surprisingly, gene coordinates did not overlap for 3,523 pairs although the same Entrez-Gene ID was assigned. Among those gene pairs for which both NCBI and Ensembl had an assigned gene symbol (8,093 pairs) or gene product name (10,188 pairs), 19% and 30% were assigned inconsistent gene symbols or gene product names, respectively. Therefore, finding a consensus gene set on the basis of multiple criteria and a sequential matching process is necessary and more reliable than using a single criterion.

Even using the above process, there were several cases for which matching "gene type" produced an unreliable result. For example, some protein coding genes in Ensembl were classified as 'pseudogenes' in NCBI, and a total of 54 genes in NCBI had the "gene type" of 'unknown' or 'other', which were not present in Ensembl gene classifications. In the amalgamated cattle database, 10 and 17 genes that were classified as 'pseudogenes' and 'unknown' or 'other', respectively; in NCBI they were found to be involved in enzymatic reactions. These were manually reclassified as protein coding genes and merged with the corresponding Ensembl genes. More unidentified consensus genes may be present in our database due to differences in "gene type" annotations in the Ensembl and NCBI databases.

We developed an amalgamated genome database that includes all the gene models predicted by Ensembl and NCBI. Our approach has several advantages. First, the amalgamated database likely contains most cattle genes. The Ensembl and NCBI gene models predict genes that are independently supported by multiple lines of biochemical and computational evidence. Therefore, there is presently insufficient evidence to reject the presence of genes predicted by either source. An amalgamated gene prediction set is thus expected to be more complete. For example, among those genes that were identified to encode enzymes for the known reactions in our database, 112 and 79 genes were predicted exclusively by Ensembl or NCBI, respectively. Another advantage of the amalgamation approach is that all available functional annotations of cattle genes can be easily incorporated into the final product, because the additional step of informatically linking IDs of the consensus gene set to KEGG, UniProt and other databases is not necessary.

### Metabolic reconstruction of the cattle genome

Although there are several bioinformatic platforms that could be used in reconstruction of genome-scale organism-specific metabolic networks, Pathway Tools has advantages over others in that 1) the Pathway Tools software allows automated and user-friendly generation of an organism-specific pathway database. PathoLogic permits mapping the functional annotation of gene products into MetaCyc, one of the largest, most comprehensive and well-curated databases for biochemical pathways; 2) currently, more than 260 PGDBs have been generated using Pathway Tools and the common 'Cyc' database format, which provides a consistent platform for the comparative analysis of metabolic pathways among different species [15]; 3) the Pathway Tools Omics Viewer can incorporate transcriptomic, proteomic, metabolomic and reaction flux data into the PGDB. It is one of the few tools that allow integration of metabolic and gene-regulatory networks [69], and 4) according to Poolman *et al*. [70], metabolic networks computationally generated from MetaCyc had lower errors (e.g. unbalanced reactions and orphan and dead-end metabolites) than those generated from KEGG. This may be an important feature if the reconstructed metabolic network is to be further applied to systems biology.

Despite these strengths, the reconstruction of metabolic networks using Pathway Tools also has some limitations. As the automated reconstruction procedure is done by linking reactions and pathways to annotated genes, the quality of such an automatically generated metabolic network highly depends on the primary genome annotation [71]. At present, functions of most mammalian genes are poorly understood and their annotations are heavily dependent on sequence homology to human and mouse [72]. This may lead to limitations in generating species-specific metabolic networks in mammals. Moreover, due to the lack of consensus in gene annotations among databases, the amalgamation of functional annotation from different sources is required.

Another pitfall of the automated reconstruction using Pathway Tools software is that many inappropriate pathways may appear in the reconstructed metabolic network. Accordingly, the initial reconstruction needs to be manually curated in a time and labor intensive manner even though a semi-automated approach to accelerate the reconstruction process has been developed [71]. This is mainly because the PathoLogic algorithm was designed to import as many candidate metabolic pathways from MetaCyc as possible for a given gene set, assuming that manual curation is done afterward [16]. Using PathoLogic is thus a conservative way of reducing the risk of missing pathways in a genome with an additional payoff in saved time and labor. Furthermore, the collection of pathways in MetaCyc is mainly from bacteria [17]. Consequently, a large proportion of predicted metabolic pathways are bacteria-specific that need to be deleted from the automated reconstruction of eukaryotes unless there is overwhelming evidence to the contrary. For example, it has been reported that 126 pathways were deleted and 17 pathways were manually reconstructed after the initial automated generation of metabolic pathways of *Medicago truncatula *[15]. Likewise, in the present study 53% of pathways in the initial automated reconstruction from cattle PGDB needed to be deleted or modified. For example, we manually modified 64 mammalian-specific metabolic pathways from the reference pathways in MetaCyc and 2 pathways (Ketogenesis and Ketone degradation) that are important in lipid metabolism of mammals [73] were added [see Additional file 2]. Although comprehensive literature searches and experimentation are the only ways to resolve false-positives, the addition of mammalian metabolic pathways reconstructed in this study into MetaCyc will reduce the effort needed to reconstruct metabolic pathways for other mammals.

Another possible way to reduce false-positives in metabolic reconstructions is to categorize pathways in the MetaCyc database on the basis of taxonomy and then to use this information for the computational reconstruction. For example, peptidoglycan is a unique polymer that forms an external layer of bacterial plasma membranes [74]. PathoLogic predicts the presence of the peptidoglycan biosynthesis pathway in mammals, and HumanCyc contains this pathway. Similarly, HumanCyc contains some of the pathways involved in biosynthesis of the hemi-cellulose components (e.g. rhamnose and arabinose) of plants [see Additional file 3]. Classification of known metabolic pathways that are taxa-specific, and incorporation of a selection option into PathoLogic, may reduce the time needed for manual curation and increase the quality of the automated reconstructions.

Missing enzymes and metabolic pathways were identified using comparative analysis after automated generation of the new PGDBs for annotated mammalian genomes (human, mouse and dog). Comparative analysis of metabolic pathways allowed us to identify unpredicted metabolic pathways of cattle using the automated procedures and to annotate functions to cattle genes in a metabolism-centered way. For example, if a metabolic pathway is present in both cattle and human, a gene involved in an enzymatic reaction in the human pathway is more likely present in cattle, and the cattle protein that has the highest sequence homology to the orthologous human protein is likely to mediate the reaction. This approach is expected to facilitate functional annotation of poorly annotated genomes with greater reliability.

Comparative analysis of metabolic pathways demonstrated that some metabolic pathways are highly conserved at both the enzyme and functional levels in cattle and *E. coli *[see Additional file 4]. Most highly conserved pathways are related to nucleotide/nucleoside metabolism and energy metabolism, which are among the most ancient [75,76]. Some differences in enzymes in the same functional pathway are related to compartmentation and localization. For example, gluconeogenesis in mammals occurs in two compartments, cytosol and mitochondria, and due to the absence of phosphoenolpyruvate synthase (EC 2.7.9.2), conversion of pyruvate directly to phosphoenolpyruvate does not occur [76]. Instead, pyruvate is converted to oxaloacetate in mitochondria, where oxaloacetate is decarboxylated into phosphoenolpyruvate by phosphoenolpyruvate carboxykinase (EC 4.1.1.32) [76]. Distribution of phosphoenolpyruvate carboxykinase between the cytosol and mitochondria may vary among mammals [76]. Clearly, if metabolism is to be modeled in higher organisms with precision, the differences in compartmentation of metabolic reactions in plants, animals and microbes must be better understood.

Although CattleCyc shares only 54% of metabolic pathways with HumanCyc (Figure 3), in actuality, few metabolic differences exist between the two species at the enzyme level on the basis of gene orthology. Upon identifying and filling pathway holes in the reconstructed cattle metabolic pathways, we found only five missing cattle orthologs of human genes in the current cattle genome. This may imply recent metabolic adaptations specific to ruminant artiodactyls. Thus, the differences in metabolism among mammals cannot be fully represented by a genome-scale global metabolic reconstruction. Nevertheless, comparative metabolic pathway analysis establishes the foundation for studying the evolution of metabolism and for directing hypothesis-driven research aimed at filling pathway holes.

We did not find evidence for the existence of mammalian genes encoding 22 metabolic enzymes for which activity was reported in the literature. There are two explanations for these results: 1) incomplete functional annotation of mammalian genomes and 2) contamination of samples with enzymes originating from other compartments of the cell or non-mammals. With respect to the first explanation, even for the human genome, more than 40% of proteins have not been functionally annotated [77]. Compounding the problem, experimental evidence for metabolic pathways tends to be skewed against less-studied metabolism [78]. Thus, incomplete annotation is likely to be the major reason for missing enzymes in metabolic pathways. Our work clearly identifies and carefully presents mammalian metabolic pathways and enzymatic reactions that require experimental validation.

The 'ascorbate biosynthesis' pathway was further investigated as an example of the "missing enzyme" problem (see [79] for a recent review of ascorbate metabolism). Except for primates and guinea pigs, which have lost their ability for ascorbate synthesis due to a highly mutated gene for L-gulonolactone oxidase [80,81], most mammals are able to synthesize ascorbate [73]. However, no mammalian genes have been identified for the four enzymes in the pathway (Tables 4 and 5). Thus, there is no genetic evidence for enzymes that catalyze the formation of L-gulono-1,4-lactone from D-glucuronate in mammals. A comprehensive literature search revealed that *RGN *(regucalcin; also known as senescence marker protein 30), which regulates calcium signaling in the liver cell [82], also has gluconolactonase activity (EC 3.1.1.17) [83]. However, there is no functional annotation of *RGN *for this catalytic activity in the NCBI, Ensembl, UniProt or KEGG databases.

An example of enzyme contamination can be also found in the ascorbate biosynthesis pathway. Two routes have been suggested for formation of D-glucuronate from UDP-glucuronate in mammals [79], either through a formation of D-glucuronate 1-phosphate or β-D-glucuronide. However, the observed pyrophosphatase activity of rat liver microsomes [84] was likely the result of contamination of the microsomal fraction with plasma membrane fragments [79]. Instead of the above intermediates, Linster and Schaftingen [85] concluded that UDP-glucuronate is directly hydrolyzed to D-glucuronate by a UDP-glucuronidase. These authors also suggested that UDP-glucuronidase may be an isoform of UDP-glucuronosyltransferase. CattleCyc assumes that D-glucuronate forms through β-D-glucuronide as an intermediate because UDP-glucuronidase has not yet been fully characterized and there is sufficient evidence that UDP-glucuronosyltransferase is involved in the formation of D-glucuronate [79,86]. These results show that a metabolism-centered comparative analysis of metabolic pathways is helpful in identifying and evaluating present gaps in our knowledge. A well-curated PGDB like CattleCyc will facilitate computational reconstruction of metabolic pathways for other mammalian genomes with greater reliability.

## Conclusion

A step-wise comprehensive approach was used for the reconstruction of metabolic pathways of cattle. An amalgamated cattle genome database was developed from two major independent annotation sources, Ensembl and NCBI, with incorporation of all the available information for proteins, mainly in UniProt and KEGG. Metabolic pathways were computationally reconstructed by matching functional annotations of genes to a well-curated biochemical pathways database (MetaCyc). Missing enzymes and pathways were identified using comparative analysis and manual curation of the automated reconstruction on the basis of comprehensive literature searches. Thus we show that a metabolism-centered comparative analysis of metabolic pathways is helpful in identifying and evaluating present gaps in our knowledge. However, metabolic pathway analysis strongly reflects the quality of the current genome annotations and knowledge of compartmentalization of metabolic enzymes and functions. Thus, although most metabolic pathways are shared between cattle and human at the genomic level, a genome-scale global metabolic reconstruction does not fully represent the true metabolic differences between these species. Differences in metabolism among mammals may result from tissue- and organelle-specific transcriptional regulation and post-transcriptional control mechanisms. Nevertheless, comparative metabolic pathway analysis is a powerful tool for studying the evolution of metabolic genes and pathways. The new CattleCyc database will contribute to understanding the evolution of mammalian metabolism and the physiology of ruminants at the systems level.

## Methods

### Development of an amalgamated genome annotation database

The NCBI cattle reference build 3.1 and the Ensembl release 43 build 3.1 were retrieved using Entrez-Gene [87] and BioMart [88], respectively, on March 2, 2007. The two data sources were separately used to provide gene models and basal annotations for the cattle genome. To incorporate all the known protein names and synonyms as well as EC numbers of gene products, the UniProt accessions and the Kyoto Encyclopedia of Genes and Genomes (KEGG) identification (ID) numbers (same as Entrez-Gene ID in most cases) available for each annotated genome were matched against those in data flat files retrieved via FTP on March 2, 2007 from UniProt Knowledgebase release 9.7 [89] and the KEGG Genome Database release 41.0 [90], respectively.

The above NCBI- and Ensembl-based comprehensive cattle genome databases were then integrated in order to eliminate redundancy, and the amalgamated genome database was used to generate input files for running PathoLogic (see below). For integration, a sequential matching process was done for all gene pairs that shared a common (partial or complete) chromosome location, including those on unassigned contigs. For gene models that had the same strand and "gene type" (i.e. protein coding, pseudogene, tRNA and miscellaneous RNA), two genes were assumed to be identical if and only if one or more of the following conditions was met: 1) gene coordinates were exactly the same, 2) gene names or synonyms were matched, 3) Entrez-Gene ID (NCBI) was matched in Ensembl, 4) gene product names were matched, and 5) EC numbers that are assigned to the genes were matched. The matching criteria in the order given above were used as a regressive scale of confidence in identifying matches. Matched genes in NCBI and Ensembl were combined into one gene under the Entrez-Gene ID, with all the associated annotations incorporated. The smaller coordinate of the two transcription start sites and the larger of the two transcription termination sites were assumed as the start and end coordinates of the final gene model, respectively. A complete amalgamated cattle genome database containing all annotations from different sources was created to facilitate the name matching process during automated reconstruction. For comparison, the same procedures were used to generate amalgamated databases for human (build 36), mouse (build 36) and dog (build 2).

### Reconstruction of metabolic pathways

Cattle metabolic pathways were reconstructed from the amalgamated cattle genome database by generating a cattle-specific PGDB using Pathway Tools 11.0. The initial cattle PGDB was then manually curated using a comparative analysis approach, which included comparison of metabolic pathways with other organisms. A generalized scheme for the metabolism-centered approach is presented in Figure 1. EcoCyc [13] 11.0 and HumanCyc [9] 11.0 were used as standards for determining the presence of pathways and enzymes, and new PGDBs for human, mouse and dog were constructed using the same procedures as described above for the cattle genome. These automated metabolic reconstructions of the human, mouse and dog genomes, which were built from the identical amalgamation process and Pathway Tools, were also compared with the cattle PGDB. For each pathway predicted in any species-specific PGDB, biochemical evidence in the literature was searched manually to determine if the pathway is present in mammals. A pathway was deleted from CattleCyc after comprehensive literature search if 1) either the input or the output of the pathway is not present in any mammal (e.g. peptidoglycan biosynthesis), or 2) neither enzyme activity was reported nor homologs were identified in any mammal, and an alternative pathway exists with strong biochemical evidence (e.g. putrescien degradation I). When a pathway present in mammals was not adequately represented in MetaCyc, it was modified on the basis of information from KEGG, the literature, and biochemistry text books. Three data sources were used intensively as references [73,76,91]. "Missing" metabolic proteins for which no gene was identified in a cattle pathway were searched for in the cattle genome and non-redundant protein databases using TBLASTN and BLASTP [92], respectively. The thresholds used for identification of the cattle ortholog of a mammalian protein are 80% coverage and 70% identity, which were similar to those used in the Ensembl gene annotation [72]. Additional orthologs were assigned if the best BLAST hit included >50% and exactly matched >90% of the query protein sequence. Reactions mediated by those enzymes were also searched for in the literature and BRENDA [93]. The bioinformatic and biochemical evidence used for gene annotation were referenced and documented in CattleCyc on the appropriate pages (e.g., protein pages and pathway pages). Manual curation of the human, mouse and dog PGDBs was not performed because this was beyond the scope of the present work. Comparative metabolic analysis was done for CattleCyc, EcoCyc and HumanCyc using the web-server interface function of PathwayTools followed by manual inspection to identify metabolic differences among these species.

## Availability and requirements

CattleCyc is freely accessible at 

## Abbreviations

PGDB: pathway genome database; EC: enzyme commission.

## Authors' contributions

SS participated in the design of the study, developed the database, reconstructed metabolic pathways of mammalian genomes, conducted manual curation and comparative analyses, and wrote the manuscript. HAL supervised the research, participated in the design of the study, and wrote the manuscript. Both authors have read the manuscript, provided critical reviews, and approved the final manuscript.

## Note added in Proof

The most recent release of Human Cyc (12.0) has many pathways that have been deleted for insufficient evidence, thus supporting our manual review and curation procedures. In addition, version 11.5 of PathoLogic incorporates taxonomy-based pathway scoring as suggested in the Discussion.

## Supplementary Material

Additional file 1 **Supplementary Table one.** Consensus gene pairs in the NCBI and Ensembl databases for selected mammalian genomes. Click here for file

Additional file 2 **Supplementary Table two.** List of pathways created in CattleCyc. Click here for file

Additional file 3 **Supplementary Table three.** HumanCyc pathways that are not incorporated in CattleCyc. Click here for file

Additional file 4 **Supplementary Table four.** Pathways shared between cattle and *E. coli*. Click here for file
