# Supplementary material for: Reconstruction of metabolic pathways for the cattle genome
Source: BMC Syst Biol. 2009 Mar 12;3:33. doi: 10.1186/1752-0509-3-33 (PMC2669051; doi:10.1186/1752-0509-3-33)
Supplement: Additional file 1 — Supplementary Table one. Consensus gene pairs in the NCBI and Ensembl databases for selected mammalian genomes. [file 1752-0509-3-33-S1.doc]

**Supplementary Table 1 - Consensus gene pairs in the NCBI and Ensembl databases for selected mammalian genomes^*^**

| Genomes | No. genes | No. (%) consensus genes |
| --- | --- | --- |
| Cattle | 37,691 | 16,173 (43) |
| Human | 41,483 | 19,354 (47) |
| Mouse | 37,610 | 20,118 (54) |
| Dog | 28,768 | 14,147 (49) |

^*^Consensus gene pairs were identified using the database amalgamation process described in Methods
