# Supplementary material for: Reconstruction of metabolic pathways for the cattle genome
Source: BMC Syst Biol. 2009 Mar 12;3:33. doi: 10.1186/1752-0509-3-33 (PMC2669051; doi:10.1186/1752-0509-3-33)
Supplement: Additional file 2 — Supplementary Table two. List of pathways created in CattleCyc. [file 1752-0509-3-33-S2.doc]

**Supplementary Table 2 - List of pathways created in CattleCyc**

| Pathway Class | Pathway^*^ | CattleCyc ID |
| --- | --- | --- |
| Biosynthesis - Amines and Polyamines | glycine betaine biosynthesis (from choline) mammals | PWY3FA-16 |
| ″ | glycine betaine biosynthesis (from glycine) mammals | PWY3FA-283 |
| ″ | UDP-N-acetyl-D-glucosamine biosynthesis mammals | PWY3FA-351 |
| Biosynthesis - Amino acids Biosynthesis | proline biosynthesis V (from arginine) mammals | PWY3FA-647 |
| ″ | s-adenosyl-L-methionine cycle mammals | PWY3FA-342 |
| ″ | tyrosine biosynthesis mammals | PWY3FA-25 |
| ″ | β-alanine biosynthesis II mammals | PWY3FA-719 |
| Biosynthesis – Carbohydrates | gluconeogenesis mammals | PWY3FA-484 |
| ″ | UDP-D-glucuronate biosynthesis (from myo-inositol) mammals | PWY3FA-3 |
| Biosynthesis - Cofactors, Prosthetic Groups, Electron Carriers | ascorbate biosynthesis mammals | PWY3FA-1894 |
| ″ | folate transformations mammals | PWY3FA-916 |
| ″ | formylTHF biosynthesis II mammals | PWY3FA-70 |
| ″ | NAD biosynthesis I (from aspartate) mammals | PWY3FA-787 |
| ″ | NAD biosynthesis II (from tryptophan) mammals | PWY3FA-518 |
| ″ | NAD salvage pathway II mammals | PWY3FA-386 |
| ″ | NAD/NADH phosphorylation and dephosphorylation mammals | PWY3FA-63 |
| ″ | pyridine nucleotide cycling mammals | PWY3FA-317 |
| ″ | riboflavin metabolism mammals | PWY3FA-26819 |
| ″ | s-adenosylmethionine biosynthesis mammals | PWY3FA-69 |
| ″ | tetrahydrofolate biosynthesis I mammals | PWY3FA-247 |
| ″ | tetrahydrofolate biosynthesis II mammals | PWY3FA-1062 |
| Biosynthesis – Fatty Acids and Lipids | ketogenesis | PWY3FA-941 |
| ″ | phospholipid biosynthesis II mammals | PWY3FA-883 |
| Biosynthesis - Nucleosides and Nucleotides | de novo biosynthesis of pyrimidine deoxyribonucleotides mammals | PWY3FA-768 |
| ″ | purine nucleotides de novo biosynthesis I mammals | PWY3FA-34 |
| ″ | salvage pathways of adenine, hypoxanthine, and their nucleosides mammals | PWY3FA-366 |

**Supplementary Table 2 – Continued**

| Biosynthesis - Nucleosides and Nucleotides | salvage pathways of guanine, xanthine, and their nucleosides mammals | PWY3FA-361 |
| --- | --- | --- |
| ″ | salvage pathways of purine and pyrimidine nucleotides mammals | PWY3FA-598 |
| ″ | salvage pathways of purine nucleosides mammals | PWY3FA-1109 |
| ″ | salvage pathways of pyrimidine deoxyribonucleotides mammals | PWY3FA-477 |
| ″ | salvage pathways of pyrimidine ribonucleotides mammals | PWY3FA-28 |
| Biosynthesis – Other | 2-methylbutyrate biosynthesis mammals | PWY3FA-509 |
| Degradation/Utilization/Assimilation – Alcohols | oxidative ethanol degradation mammals | PWY3FA-597 |
| Degradation/Utilization/Assimilation – Aldehydes | methylglyoxal degradation mammals | PWY3FA-111 |
| Degradation/Utilization/Assimilation – Amines and Polyamines | N-acetylglucosamine , N-acetylmannosamine and N-acetylneuraminic acid dissimilation mammals | PWY3FA-491 |
| Degradation/Utilization/Assimilation – Amino Acids | 4-hydroxyproline degradation I mammals | PWY3FA-596 |
| ″ | citrulline degradation mammals | PWY3FA-448 |
| ″ | glutamate degradation IV mammals | PWY3FA-601 |
| ″ | glutamine degradation II mammals | PWY3FA-240 |
| ″ | histidine degradation III mammals | PWY3FA-639 |
| ″ | isoleucine degradation I mammals | PWY3FA-98 |
| ″ | leucine degradation I mammals | PWY3FA-5 |
| ″ | lysine degradation VII mammals | PWY3FA-1613 |
| ″ | tryptophan degradation I (via anthranilate) mammals | PWY3FA-612 |
| ″ | tryptophan degradation III (eukaryotic) mammals | PWY3FA-899 |
| ″ | tryptophan degradation VI (via tryptamine) mammals | PWY3FA-742 |
| ″ | valine degradation I mammals | PWY3FA-1237 |
| Degradation/Utilization/Assimilation – Carbohydrates | galactose assimilation III mammals | PWY3FA-144 |
| ″ | mannose degradation mammals | PWY3FA-2 |
| ″ | starch degradation mammals | PWY3FA-310 |
| ″ | sucrose degradation mammals | PWY3FA-143 |
| Degradation/Utilization/Assimilation – Carboxylates | N-acetylneuraminate degradation mammals | PWY3FA-908 |

**Supplementary Table 2 – Continued**

| Degradation/Utilization/Assimilation – Carboxylates | pyruvate degradation II mammals | PWY3FA-798 |
| --- | --- | --- |
| Degradation/Utilization/Assimilation – Fatty Acids and Lipids | fatty acid α-oxidation mammals | PWY3FA-15 |
| ″ | glutaryl-CoA degradation mammals | PWY3FA-52 |
| ″ | glycerol degradation IV mammals | PWY3FA-230 |
| ″ | ketone degradation | PWY3FA-1022 |
| ″ | sphingolipid metabolism mammals | PWY3FA-563 |
| ″ | triacylglycerol degradation mammals | PWY3FA-17 |
| Degradation/Utilization/Assimilation – Nucleosides and Nucleotides | (deoxy)ribose phosphate degradation mammals | PWY3FA-824 |
| ″ | degradation of purine deoxyribonucleosides mammals | PWY3FA-498 |
| ″ | degradation of pyrimidine deoxyribonucleosides mammals | PWY3FA-1111 |
| ″ | purine degradation mammals | PWY3FA-1699 |
| Degradation/Utilization/Assimilation – Other | octane oxidation mammals | PWY3FA-506 |
| Generation of precursor metabolites and energy | glycolysis mammals | PWY3FA-485 |
| ″ | TCA cycle mammals | PWY3FA-4 |

^*^Pathway names were modified from the name in MetaCyc by adding ‘mammals’ at the end, if applicable. The class of pathways follows the original pathway classification in MetaCyc.
