# Supplementary material for: Reconstruction of metabolic pathways for the cattle genome
Source: BMC Syst Biol. 2009 Mar 12;3:33. doi: 10.1186/1752-0509-3-33 (PMC2669051; doi:10.1186/1752-0509-3-33)
Supplement: Additional file 3 — Supplementary Table three. HumanCyc pathways that are not incorporated in CattleCyc. [file 1752-0509-3-33-S3.doc]

**Supplementary Table 3 – HumanCyc pathways that are not incorporated in CattleCyc**

| Pathways in HumanCyc | Modification needed in | Notes |
| --- | --- | --- |
| 2-oxobutanoate degradation I | CattleCyc | Possible pathway in mammals |
| cholesterol biosynthesis III (via desmosterol) | CattleCyc | Possible pathway in mammals |
| glutamate degradation I | CattleCyc | Possible pathway in mammals |
| glutamate degradation VII | CattleCyc | Possible pathway in mammals |
| L-cysteine degradation VI | CattleCyc | Possible pathway in mammals |
| oxidative ethanol degradation III | CattleCyc | The first enzyme is missing in the CattleCyc pathway. CattleCyc should incorporate it |
| Fatty acid elongation -- unsaturated I | HumanCyc and CattleCyc | This pathway should be modified and then incorporated into CattleCyc |
| glycolysis II | HumanCyc | Conversion of beta-D-Glucose to beta-D-glucose-6-phosphate (bacterial); bacterial pathway |
| glycolysis III | HumanCyc | Closer to mammalian pathway but it is not a good representation; no possible conversion of 1,3-diphosphoateglycerate to 3-phosphoglycerate via 2,3-diphosphoglycerate; no reverse from pyruvate to phosphoenolpyruvate |
| glucose and glucose-1-phosphate degradation | HumanCyc | The pathway does not represent well what happens in mammals |
| histidine degradation I | HumanCyc | The pathway does not represent well what happens in mammals |
| isoleucine degradation I | HumanCyc | The pathway does not represent well what happens in mammals |
| glyceraldehyde 3-phosphate degradation | HumanCyc | The pathway does not represent well what happens in mammals. A redundant pathway as it is a part of glycolysis. |
| lactate oxidation | HumanCyc | The pathway does not represent well what happens in mammals; but incorporation of modified version might be useful in CattleCyc |
| leucine degradation I human | HumanCyc | The pathway does not represent well what happens in mammals; the same reactions but reverse order (acetoacetate part) has been incorporated into CattleCyc |

**Supplementary Table 3 – Continued**

| Fatty acid elongation -- unsaturated II | HumanCyc | No enzymes has been identified |
| --- | --- | --- |
| GDP-D-rhamnose biosynthesis | HumanCyc | Insufficient evidence of its presence in mammals |
| GDP-mannose metabolism | HumanCyc | Insufficient evidence of its presence in mammals |
| glyoxylate cycle | HumanCyc | Insufficient evidence of its presence in mammals |
| lactose degradation II | HumanCyc | Insufficient evidence of its presence in mammals |
| mandelate degradation I | HumanCyc | Insufficient evidence of its presence in mammals |
| methionine and methyl-donor-molecule biosynthesis | HumanCyc | Insufficient evidence of its presence in mammals |
| methionine biosynthesis I | HumanCyc | Insufficient evidence of its presence in mammals |
| methionine salvage pathway I | HumanCyc | Insufficient evidence of its presence in mammals |
| O-antigen biosynthesis | HumanCyc | Insufficient evidence of its presence in mammals |
| peptidoglycan biosynthesis I | HumanCyc | Insufficient evidence of its presence in mammals |
| polyisoprenoid biosynthesis | HumanCyc | Insufficient evidence of its presence in mammals |
| protocatechuate degradation II (ortho-cleavage pathway) | HumanCyc | Insufficient evidence of its presence in mammals |
| putrescine degradation I | HumanCyc | Insufficient evidence of its presence in mammals |
| pyridoxal 5'-phosphate biosynthesis | HumanCyc | Insufficient evidence of its presence in mammals |
| pyruvate oxidation pathway | HumanCyc | Insufficient evidence of its presence in mammals |
| quinate degradation | HumanCyc | Insufficient evidence of its presence in mammals |
| reductive acetyl coenzyme A pathway | HumanCyc | Insufficient evidence of its presence in mammals |
| serine-isocitrate lyase pathway | HumanCyc | Insufficient evidence of its presence in mammals |
| shikimate degradation | HumanCyc | Insufficient evidence of its presence in mammals |
| stearoyl-ACP desaturation pathway | HumanCyc | Insufficient evidence of its presence in mammals |
| UDP-D-apiose biosynthesis (from UDP-D-glucuronate) | HumanCyc | Insufficient evidence of its presence in mammals |
| UDP-D-galacturonate biosynthesis I (from UDP-D-glucuronate) | HumanCyc | Insufficient evidence of its presence in mammals |
| UDP-D-galacturonate biosynthesis II (from D-galacturonate) | HumanCyc | Insufficient evidence of its presence in mammals |
| UDP-L-arabinose biosynthesis I (from UDP-xylose) | HumanCyc | Insufficient evidence of its presence in mammals |
| UDP-L-arabinose biosynthesis II (from L-arabinose) | HumanCyc | Insufficient evidence of its presence in mammals |
| 4-hydroxymandelate degradation | HumanCyc | Insufficient evidence of its presence in mammals |

**Supplementary Table 3 – Continued**

| acetate utilization and formation | HumanCyc | Insufficient evidence of its presence in mammals |
| --- | --- | --- |
| alanine biosynthesis I | HumanCyc | Insufficient evidence of its presence in mammals |
| arginine degradation III | HumanCyc | Insufficient evidence of its presence in mammals |
| benzoate degradation I (aerobic) | HumanCyc | Insufficient evidence of its presence in mammals |
| catechol degradation to β-ketoadipate | HumanCyc | Insufficient evidence of its presence in mammals |
| dTDP-L-rhamnose biosynthesis I | HumanCyc | Insufficient evidence of its presence in mammals |
| Entner-Doudoroff pathway II (non-phosphorylative) | HumanCyc | Insufficient evidence of its presence in mammals |
| Fatty acid biosynthesis - initial steps II (plant) | HumanCyc | Insufficient evidence of its presence in mammals |
| proline biosynthesis I | HumanCyc | Insufficient evidence of its presence in mammals; MetaCyc description also states mammals have proline biosynthesis II instead. |
| methionine biosynthesis IV | HumanCyc | A part of S-adenosylmethionine biosynthesis in CattleCyc |
| proline degradation III | HumanCyc | A redundant pathway. It is the same as proline degradation I |
| glutamate degradation IV |  | A redundant pathway as it is a part of glutamate degradation VII. |
| spermine biosynthesis II |  | A redundant pathway. In CattleCyc this pathway is done with putrescine biosynthesis III -> spermidine biosynthesis -> spermine biosynthesis |
| oxidative ethanol degradation II |  | In HumanCyc oxidative ethanol degradation is divided into three pathways. CattleCyc pathway has I and II and one more that are not incorporated into HumanCyc |
| UDP-L-rhamnose biosynthesis |  | Insufficient evidence of its presence in mammals; not in HumanCyc (v. 11.5) |
| β-ketoadipate degradation |  | Insufficient evidence of its presence in mammals; not in HumanCyc (v. 11.5) |
| UDP-N-acetylgalactosamine biosynthesis |  | Insufficient evidence of its presence in mammals; not in HumanCyc (v. 11.5): it has been combined with UDP-N-acetyl-D-glucosamine biosynthesis |
| phospholipid biosynthesis I |  | Possible pathway in mammals; Redundant pathway with phospholipid biosynthesis II; However, because of linkage it may be useful to incorporate into CattleCyc |
