# Supplementary material for: Reconstruction of metabolic pathways for the cattle genome
Source: BMC Syst Biol. 2009 Mar 12;3:33. doi: 10.1186/1752-0509-3-33 (PMC2669051; doi:10.1186/1752-0509-3-33)
Supplement: Additional file 4 — Supplementary Table four. Pathways shared between cattle and E. coli. [file 1752-0509-3-33-S4.doc]

**Supplementary Table 4 – Pathways shared between cattle and E. coli**

| Pathways | Consensus Type | No. reactions |
| --- | --- | --- |
| tRNA charging pathway | Enzyme | 20 |
| colanic acid building blocks biosynthesis | Enzyme | 10 |
| de novo biosynthesis of pyrimidine ribonucleotides | Enzyme | 10 |
| fatty acid β-oxidation I | Enzyme | 7 |
| glycogen degradation | Enzyme | 7 |
| degradation of purine ribonucleosides^†^ | Enzyme | 6 |
| fatty acid biosynthesis - initial steps | Enzyme | 6 |
| aerobic respiration -- electron donors reaction list^‡^ | Enzyme | 5 |
| coenzyme A biosynthesis | Enzyme | 5 |
| fatty acid elongation – saturated | Enzyme | 5 |
| folate polyglutamylation I | Enzyme | 5 |
| pentose phosphate pathway (non-oxidative branch) | Enzyme | 5 |
| pyridoxal 5'-phosphate salvage pathway | Enzyme | 5 |
| biotin-carboxyl carrier protein | Enzyme | 4 |
| UDP-galactose biosynthesis (salvage pathway from galactose using UDP-glucose) | Enzyme | 4 |
| 4-aminobutyrate degradation I^†^ | Enzyme | 3 |
| degradation of pyrimidine ribonucleosides^†^ | Enzyme | 3 |
| formaldehyde oxidation II (glutathione-dependent) | Enzyme | 3 |
| glycerol degradation I | Enzyme | 3 |
| glycine cleavage complex | Enzyme | 3 |
| lipoate biosynthesis and incorporation I | Enzyme | 3 |
| methylmalonyl pathway | Enzyme | 3 |
| N-acetyl-glucosamine degradation | Enzyme | 3 |
| pentose phosphate pathway (oxidative branch) | Enzyme | 3 |
| pyruvate dehydrogenase complex | Enzyme | 3 |
| selenocysteine biosynthesis^†^ | Enzyme | 3 |
| serine biosynthesis^†^ | Enzyme | 3 |
| arginine degradation III (arginine decarboxylase/agmatinase pathway) | Enzyme | 2 |
| asparagine biosynthesis III | Enzyme | 2 |
| glutathione biosynthesis | Enzyme | 2 |
| glutathione redox reactions II | Enzyme | 2 |
| lipoate biosynthesis and incorporation II^†^ | Enzyme | 2 |
| proline degradation I | Enzyme | 2 |
| putrescine biosynthesis I | Enzyme | 2 |
| removal of superoxide radicals | Enzyme | 2 |
| spermidine biosynthesis | Enzyme | 2 |
| thioredoxin pathway | Enzyme | 2 |
| threonine degradation II | Enzyme | 2 |
| trehalose degradation II (high osmolarity) | Enzyme | 2 |
| alanine biosynthesis II | Enzyme | 1 |
| alanine biosynthesis III | Enzyme | 1 |
| aspartate biosynthesis I | Enzyme | 1 |
| glycine biosynthesis I | Enzyme | 1 |

**Supplementary Table 4 – Continued**

| lactose degradation III | Enzyme | 1 |
| --- | --- | --- |
| L-cysteine degradation II | Enzyme | 1 |
| L-serine degradation | Enzyme | 1 |
| PRPP biosynthesis I | Enzyme | 1 |
| putrescine biosynthesis III^‡^ | Enzyme | 1 |
| ribose degradation | Enzyme | 1 |
| purine nucleotides de novo biosynthesis I | Function | 14 |
| de novo biosynthesis of pyrimidine deoxyribonucleotides^†^ | Function | 13 |
| Gluconeogenesis | Function | 13 |
| formylTHF biosynthesis I | Function | 12 |
| salvage pathways of adenine, hypoxanthine, and their nucleosides^†^ | Function | 12 |
| tetrahydrofolate biosynthesis I^*^ | Function | 12 |
| glycolysis I | Function | 11 |
| TCA cycle | Function | 10 |
| N-acetylglucosamine, N-acetylmannosamine and N-acetylneuraminic acid dissimilation | Function | 9 |
| salvage pathways of pyrimidine ribonucleotides | Function | 8 |
| degradation of purine deoxyribonucleosides^†^ | Function | 7 |
| salvage pathways of guanine, xanthine, and their nucleosides | Function | 7 |
| degradation of pyrimidine deoxyribonucleosides^*^ | Function | 6 |
| NAD biosynthesis I (from aspartate) ^†^ | Function | 6 |
| salvage pathways of pyrimidine deoxyribonucleotides^†^ | Function | 5 |
| UDP-N-acetyl-D-glucosamine biosynthesis | Function | 4 |
| NAD phosphorylation and dephosphorylation^‡^ | Function | 3 |
| S-adenosylmethionine biosynthesis | Function | 3 |
| glycine betaine biosynthesis I (Gram-negative bacteria) | Function | 2 |
| mannose degradation | Function | 2 |
| methylglyoxal degradation II | Function | 2 |

^†^Pathway contains one pathway hole (missing enzyme)

^‡^Pathway contains two pathway holes (missing enzymes)

^*^Pathway contains three or more pathway holes (missing enzymes)
